# **Main complications of epilepsy surgery performed in a single center in Latin America**

**DOI:** 10.1007/s10143-025-03665-0

**Published:** 2025-06-14

**Authors:** Irving Fuentes-Calvo, Jimena Gonzalez-Salido, Fernando Sotelo-Díaz, Jimena Colado-Martinez, Irene Gómez-Oropeza, Betsy C. Vázquez-Cruz, Fernando Vasquez-Lopez, Luis A. Marin-Castañeda, Mario A. Sebastián-Díaz, Sergio Moreno-Jiménez, Alfonso Arellano-Reynoso, Guillermo Axayacalt Gutiérrez-Aceves, Salvador Martínez-Medina, Santiago Philibert-Rosas, Oscar Isaac Vázquez-Hernández, Juan Carlos Vera-López, Nahomi M. Herrera-Noguera, Fernando M. Chavez-Hassan, Juana Villeda-Hernandez, Maximo Leon-Vazquez, Aurelio Jara-Prado, Adriana Ochoa-Morales, Jorge Guerrero-Camacho, Diego A. Barrios-González, Mario A. Alonso-Vanegas, Iris E. Martínez-Juárez

**Affiliations:** 1 Epilepsy Clinic & Clinical Epileptology Fellowship, National Institute of Neurology and Neurosurgery “Manuel Velasco Suárez” & Faculty of Medicine, UNAM, Mexico City, Mexico; 2 Neurosurgery Residency Program, National Institute of Neurology and Neurosurgery, Mexico City, Mexico; 3 Nephrology Department, South Central High Specialty Hospital PEMEX, Mexico City, Mexico; 4 Radioneurosurgery Unit, National Institute of Neurology and Neurosurgery, Mexico City, Mexico; 5 Functional Neurosurgery Department, National Institute of Neurology and Neurosurgery, Mexico City, Mexico; 6 Neurology Resident, National Institute of Neurology and Neurosurgery, Mexico City, Mexico; 7 Epilepsy Department, Hospital Bautista, Managua, Nicaragua; 8 Experimental Pathology Laboratory, National Institute of Neurology and Neurosurgery, Mexico City, Mexico; 9 Department of Neuroscience, Instituto Mexicano del Seguro Social Centro Médico Nacional “La Raza”, Mexico City, Mexico; 10 Genetics Department, National Institute of Neurology and Neurosurgery, Mexico City, Mexico; 11 General Surgery Residency Program, General Hospital, IMSS., 20 La Margarita, Puebla City, México; 12 International Epilepsy Center, HMG Coyoacán, Mexico City, Mexico; 13 Clinical Neurophysiology and Cognition Laboratory, National Institute of Neurology and Neurosurgery, Insurgentes Sur 3817, Colonia La Fama, Tlalpan, Mexico City, 14269 Mexico; 14 Grupo Neurológico, Neuroquirúrgico y de Columna. Hospital Ángeles Acoxpa, Mexico City, Mexico

**Keywords:** Epilepsy, Epilepsy surgery, Neurosurgery, Main complications, LATAM, Functional neurosurgery

## Abstract

**Objective:**

Describe the main medical and neurological complications following epilepsy surgery at a tertiary care center in Latin America.

**Materials and methods:**

A retrospective study was conducted from 2006 to 2013 at the National Institute of Neurology and Neurosurgery in Mexico City. Patients aged over 18 years with drug-resistant epilepsy who underwent surgery and had a minimum follow-up of one year were included. Statistical analyses performed were Fisher’s exact test, Pearson’s Chi-square, and one-way ANOVA with Tukey post hoc for multiple comparisons.

**Results:**

Of 204 clinical records reviewed, 165 met inclusion criteria, and 95 (57.6%) underwent epilepsy surgery. Most patients (73.7%) had temporal lobectomy with amygdalohippocampectomy, followed by lesionectomy (9.5%), corpus callosotomy (15.8%), and one (0.6%) vagus nerve stimulator implantation. Minor medical complications occurred in 6.3% of patients, with extracranial infection (4.2%) and CSF fistula (2.1%) being the most common. Minor neurological complications were observed in 29.5%, including cranial nerve deficits (2.1%), intracranial hematoma (2.1%), and quadrantanopia (25.3%). One patient (1.1%) experienced a major complication (hemianopsia).

**Conclusion:**

This large LATAM cohort highlights the low complication rate of epilepsy surgery. Early referral of DRE patients demonstrated statistically significant favorable outcomes and fewer postoperative complications. Despite its demonstrated safety when performed by experienced specialists, its underutilization persists due to access barriers, even though untreated epilepsy poses significantly greater risks.

## Introduction

Pharmacological treatment is widely recognized as the primary approach for managing epilepsy. It offers the potential to reduce seizure frequency and severity, with the ultimate goal of achieving seizure freedom in people with epilepsy (PWE). This approach provides a significant advantage due to the wide range of available antiseizure medications (ASMs), allowing for personalized treatment and minimization of adverse effects depending on the seizure types and epilepsy syndromes [1].

Approximately 60–70% of patients with focal epilepsy achieve seizure freedom with ASMs. However, the remaining patients who continue to experience seizures despite optimal pharmacological treatment are classified as drug-resistant epilepsy (DRE). For these, alternative therapies have been explored, such as epilepsy surgery, vagus nerve stimulation (VNS), deep brain stimulation (DBS), and/or ketogenic diet [2, 3].

Globally, an estimated 10.1 million individuals are candidates for epilepsy surgery, with approximately 1.4 million new surgically treatable cases identified each year. Notably, the highest burden of epilepsy coincides with regions that have the most limited access to surgical and diagnostic resources, particularly in Africa, Latin America (LATAM), and other low- and middle-income countries. *(Vaughan et al. 2019)*. In LATAM specifically, epilepsy surgery has been a growing trend over the past few decades; significant efforts have been directed toward expanding neurosurgical procedures [4]. Central to these efforts has been the adoption of a comprehensive pre-surgical evaluation and planning incorporating a neuropsychological profile to optimize surgical outcomes and minimize complications [5].

Complications from epilepsy surgery are related to the extent and location of the resected area and the surgical technique used. Main complications can be classified as: (1) Permanent, such as functional, cognitive, or neurological deterioration, often secondary to hemorrhage; (2) Transient complications that include postoperative infections and cerebrospinal fluid collections [6]. In a meta-analysis by WJ Hader et al., postoperative complications were classified into medical and neurological categories. Medical complications were further divided into minor, such as cerebrospinal fluid (CSF) leaks, intra- or extracranial infections, aseptic meningitis, pneumonia, and metabolic disorders, and major, including hydrocephalus, deep infections, and intracerebral or epidural abscesses. Neurological complications were classified into minor, temporary, and resolved completely within 3 months post-surgery, and major, which persist beyond 3 months. Neurological issues can include cranial nerve deficits, dysphasia, memory impairment, or hemiparesis. Visual field-related complications were categorized as minor if they involved quadrantanopia and major if they involved hemianopsia [7]. Moreover, neuropsychiatric follow-up is recommended, given that neuropsychiatric symptoms are prevalent among PWE, and postoperative exacerbation of these symptoms has been reported [8].

Few studies from LATAM systematically report the incidence and classification of surgical complications, and the underutilization of epilepsy surgery in this region remains a critical barrier to optimal care. Addressing this gap is essential to promote equitable access and inform regional surgical practices. Herein, we present an analysis of medical and neurological complications following epilepsy surgery performed at a single tertiary care center in LATAM.

## Materials and methods

### Patient selection

Following institutional review board approval (No. 74/13), ambispective review of medical records was performed from 2006 to 2013 and prospectively up to 2025 to identify adult patients who underwent epilepsy surgery and its postoperative complications at the National Institute of Neurology and Neurosurgery “Manuel.

Velasco Suárez” (NINN) in Mexico.

Inclusion criteria encompassed patients aged over 18 years diagnosed with either focal or generalized epilepsy who had undergone epilepsy surgery, with a minimum follow-up of at least one year. Patients received pre- and post-operative evaluations from the Epilepsy Clinic by an epileptologist (I.E.M.J.), clinical epileptology fellows, and epilepsy surgeons (M.A.V., S.M.J., A.A.R., and G.A.G.A.), and epilepsy surgery and functional fellows. Eligible patients also had EEG or video-EEG, magnetic resonance imaging (MRI), neuro-ophthalmology, neuropsychology, and psychiatry evaluations.

Patients were excluded if they did not meet the inclusion criteria, declined surgery, had undergone prior surgical procedures outside the NINN, had a follow-up period of less than six months, or lacked the required postoperative follow-up evaluations.

### Statistical analysis

SPSS^®^ version 29 was used for data entry and statistical analysis. Fisher’s exact test and Pearson’s Chi-square test were performed. A one-way ANOVA test was used for numerical variables with a normal distribution. Tukey’s post hoc test was performed for multiple comparisons to identify which groups exhibited significant differences, ensuring proper control of Type I error. The Kolmogorov test was used to confirm a normal distribution of numerical values. P-values < 0.05 were considered statistically significant.

### Postoperative complications classification

Postoperative complications identified in this analysis were categorized using the classification system proposed by W.J. Hader et al. [7]. *Medical complications* were classified as minor or major. Minor complications included CSF leaks, intracranial and extracranial infections, aseptic meningitis, pneumonia, and metabolic disorders. Major complications encompassed hydrocephalus, deep infections, intracerebral or epidural abscesses, and other neurological complications. *Neurological complications* were further categorized based on their duration. Minor complications, which resolved completely within three months post-surgery, included cranial nerve deficits, dysphasia, memory impairment, hemiparesis, and quadrantanopia. In contrast, major neurological complications persisting beyond three months included cranial nerve deficits, dysphasia, memory impairment, hemiparesis, and hemianopia.

## Results

### Patient sample selection and clinical characteristics

Clinical records of 204 patients from 2006 to 2013 were reviewed. Following a secondary screening, 165 patients met the inclusion criteria and underwent NINN´s epilepsy surgery protocol. The majority were diagnosed with temporal lobe epilepsy (TLE) (*n* = 103, 63%), followed by extratemporal lobe epilepsy (ETLE) (*n* = 35, 21.2%), Lennox-Gastaut syndrome (LGS) (*n* = 18, 10.9%), and miscellaneous epilepsy syndromes (*n* = 8, 4.8%).

Despite completing the epilepsy surgery protocol, only 95 (57.6%) patients proceeded with surgical intervention. The remaining 70 (42.4%) patients did not undergo surgery for various reasons: 15 (9.1%) declined the procedure, 39 (23.6%) were deemed non-surgical candidates during the epilepsy surgery session, and 16 (9.7%) remained on the waiting list at the time of the study.

Of the 95 PWE who underwent surgery, 50 (53%) were male and 45 (47.4%) were female. The mean age at the time of evaluation was 35.54 ± 9.68 years (range: 18–60), with a mean age at epilepsy onset of 9.33 ± 7.2 years and a mean age at the time of surgery of 32 ± 9.95 years (see Table 1).

**Table 1:** Patient’s clinical characteristics and epilepsy surgery complications

		Lobectomy *n* (%)	Callosotomy *n* (%)	Lesionectomy *n* (%)	VNS *n* (%)	Total *n* (%)	*p*
**Age (Mean ± SD)**		36.04 ± 10.357 (95% CI 34.45–37.63)					**< 0.001**
**Sex**	**Male**	36 (43.4)	10 (12)	4 (4.8)	1 (1.2)	51 (61.4)	0.509
	**Female**	34 (41.5)	5 (6.1)	5 (6.1)	0 (0)	44 (53.7)	
**Age at epilepsy onset (Mean ± SD)**		9.74 ± 7.89 (95% CI 8.53–10.96)					**< 0.001**
**Complications**	**None**	40 (66.7)	12 (20)	7 (11.7)	1 (1.7)	60 (100)	0.075
	**Medical minor**	3 (50)	2 (33.3)	1 (16.7)	0 (0)	6 (100)	
	**Neurological minor**	26 (92.9)	1 (3.6)	1 (3.6)	0 (0)	28 (100)	
	**Neurological major**	1 (100)	0 (0)	0 (0)	0 (0)	1 (100)	
**Medical minor complications**	**None**	66 (75)	13 (14.8)	8 (9.1)	1 (1.1)	88 (100)	0.248
	**CSF fistula**	1 (50)	0 (0)	1(50)	0 (0)	2 (100)	
	**Intracranial infections**	1 (100)	0 (0)	0 (0)	0 (0)	1 (100	
	**Extracranial infections**	2 (50)	2 (50)	0 (0)	0 (0)	4 (100)	
**Neurological minor**	**None**	66 (74.2)	14 (15.7)	8 (9)	1 (1.1)	89 (100)	0.177
	**Cranial nerve deficits**	2 (100)	0 (0)	0 (0)	0 (0)	2 (100)	
	**Status epilepticus**	0 (0)	1 (100)	0 (0)	0 (0)	1 (100)	
	**Intracranial hematoma**	2 (100)	0 (0)	0 (0)	0 (0)	2 (100)	
	**Dysphasia**	0 (0 )	0 (0)	1 (100)	0 (0)	1 (100)	
**Neurological major**	**None**	67 (73.6)	15 (16.5)	8 (8.8)	1 (1.1)	91 (100)	0.565
	**Cranial nerve deficits**	1 (100)	0 (0)	0 (0)	0 (0)	1 (100)	
	**Memory alterations**	1 (100)	0 (0)	0 (0)	0 (0)	1 (100)	
	**Hemiparesia**	1 (50)	0 (0)	1 (50)	0 (0)	2 (100)	
**Visual complications**	**Quadrantanopsias**	24 (100)	0 (0)	0 (0)	0 (0)	24 (100)	**0.004**
	**Hemianopsias**	1 (100)	0 (0)	0 (0)	0 (0)	1 (100)	

*Percentages in this section are calculated within each complication type (row-wise)

### MRI findings

The most common finding was unilateral hippocampal sclerosis presented in 52 (54.7%) patients, followed by cortical dysplasia in 11(11.6%) patients, and bilateral temporal hippocampal sclerosis in seven (7.3%) patients.

### Epilepsy surgery performed

Regarding the type of epilepsy surgery performed, 70 (73.7%) patients underwent a temporal lobectomy with amygdalohippocampectomy, 9 (9.5%) lesionectomy, 15 (15.8%) corpus callosotomy (CC), and VNS was implanted in one patient. Transoperative electrocorticography (ECOG) was performed in 83 (87.4%) of the total operated patients.

### Seizure outcome

Postoperative seizure outcome was determined with Engel’s seizure freedom scale where 65 (68.5%) patients were classified as Engel I, of which 37 (38.9%) patients were classified as Ia, 9 (9.5%) were classified as Engel II, 11 (11.6%) were classified as Engel III, and 10 (10.5%) were classified as Engel IV, of which only 2 (2.1%) were IVc (Table 2).

**Table 2:** Postoperative seizure outcomes measured by engel’s seizure freedom scale

Engel	*n*	%
**I**	**65**	**68.5**
Ia	37	38.9
Ib	24	25.3
Ic	1	1.1
Id	3	3.2
**II**	**9**	**9.5**
IIa	0	0
IIb	4	4.2
IIc	3	3.2
IId	2	2.1
**III**	**11**	**11.6**
IIIa	11	11.6
IIIb	0	0
**IV**	**10**	**10.5**
IVa	6	6.3
IVb	2	2.1
IVc	2	2.1
**Total**	**95**	**100**

### Antisezuire medication

Preoperatively, the majority of patients were treated with multiple ASMs. Specifically, 40 (42.1%) patients were on three ASMs, 19 (20%) on four, 5 (5.3%) on five ASMs, and another 5 (5.3%) on more than six ASMs. In contrast, only one (1.1%) patient was on monotherapy, and 25 (26.3%) received dual therapy.

Postoperative usage of ASM was assessed two years after surgery. It was observed that 2 (2.2%) patients no longer required ASMs, while 19 (20.7%) patients were maintained on monotherapy. Additionally, 26 (28.3%) patients were on dual therapy, 30 (32.6%) patients remained on three ASMs, 11 (12%) patients were on four ASMs, and 4 (4.3%) patients continued with five ASMs.

Preoperatively, the most commonly used ASM was carbamazepine, prescribed to 58 (61.1%) patients, followed by VPA in 53 (55.8%) patients, lamotrigine in 42 (44.2%) patients, and phenytoin in 25 (26.3%) patients. Post-operatively, carbamazepine remained the most frequently used medication in 39 (41.1%) patients, followed by VPA in 31(32.6%) patients, lamotrigine in 30 (31.6%) patients, and phenytoin in 15 (15.8%) patients (Table 3).

**Table 3:** Antiseizure medications prescribed pre- and post-operatively

ASMs	Before Surgery *n* (%)	After Surgery *n* (%)
**Carbamazepine**	58 (61.1)	39 (41.1)
**Valproate**	53 (55.8)	31 (32.6)
**Lamotrigine**	42 (44.2)	30 (31.6)
**Topiramate**	27 (28.4)	18 (18.9)
**Levetiracetam**	26 (27.4)	28 (29.5)
**Clonazepam**	26 (27.4)	18 (18.9)
**Phenytoin**	25 (26.3)	15 (15.8)
**Oxcarbazepine**	13 (13.7)	14 (14.7)
**Clobazam**	11 (11.6)	15 (15.8)
**Primidone**	9 (9.5)	5 (5.3)
**Pregabalin**	9 (9.5)	6 (6.3)
**Gabapentin**	4 (4.2)	2 (2.1)
**Lacosamide**	1 (1.1)	2 (2.1)
**Diazepam**	1 (1.1)	0 (0)

### Epilepsy surgery complications (Table 1; Fig. 1)

#### Medical

##### Medical minor

Six (6.3%) patients experienced minor medical complications, the most prevalent of which was extracranial infection, which occurred in four (4.2%) patients, while two (2.1%) patients developed CSF fistula.

##### Medical major

No major medical complications secondary to any type of epilepsy surgery were observed in this cohort.

#### Neurological

##### Neurological minor

According to their timing and postoperative course, minor neurological complications were observed in 28 (29.5%) patients, of which 2 (2.1%) patients developed cranial nerve deficits, 2 (2.1%) intracranial hematomas, and one (1.1%) patient presented dysphasia and another status epilepticus. Visual field complications were observed in 25 (26.3%) patients, of which 24 (25.3%) exhibited quadrantanopsia.

##### Neurological major

One (1.1%) patient presented major neurological complications, including cranial nerve deficits, memory disturbances, hemiparesis, and hemianopsia.

**Fig. 1:**
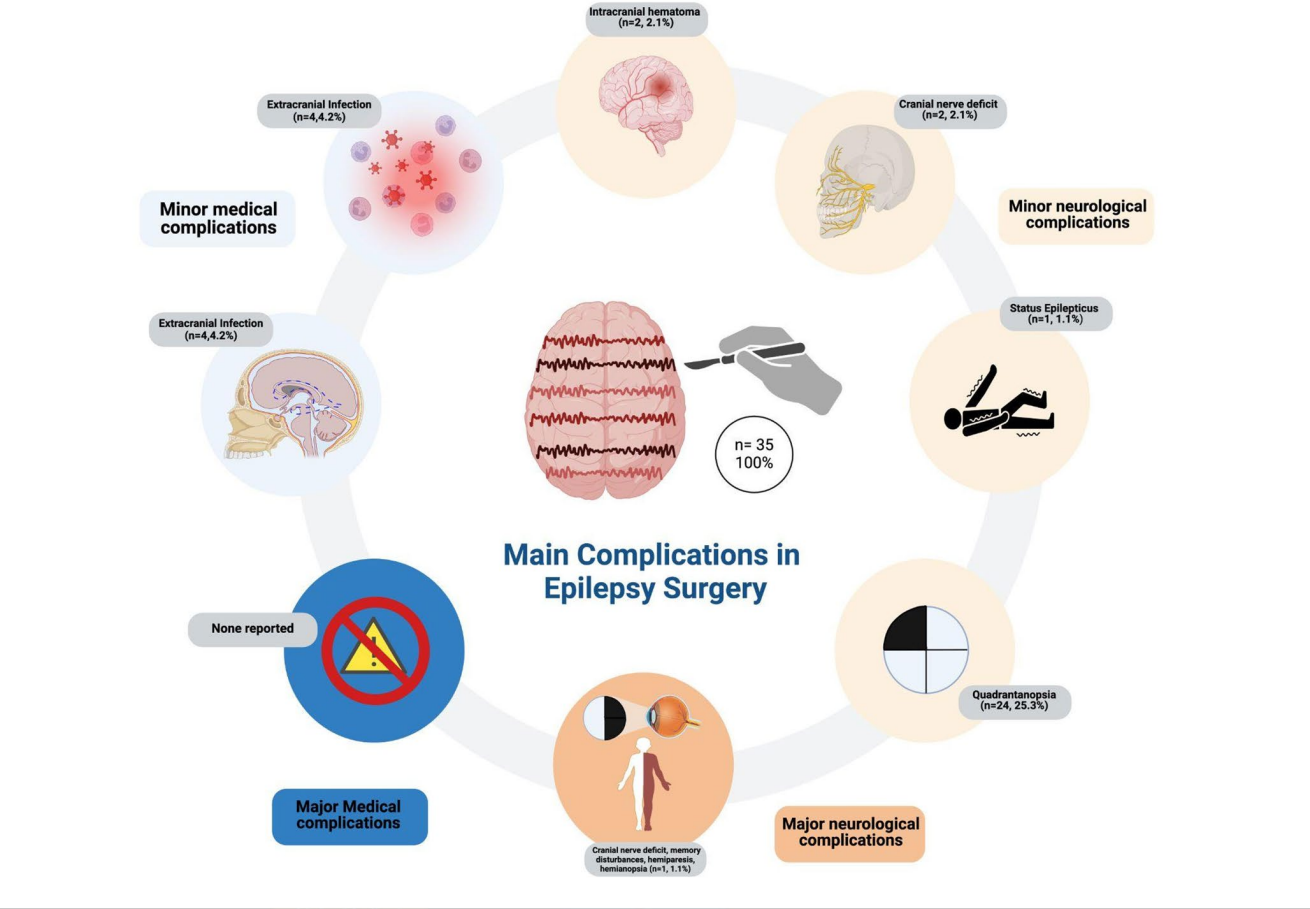
Main neurological and systemic complications in epilepsy surgery

## Discussion

### Epilepsy surgery outcome regarding medical and neurological complications

The findings in this cohort indicate a low complication rate related to epilepsy surgeries performed in our center, along with favorable postoperative seizure outcomes. Most complications in this cohort were attributed to lobectomies, followed by CC and, subsequently, lesionectomy.

In this series, only six (6.3%) patients experienced minor medical complications, and none had major ones. Additionally, only one-third of the patients had minor neurological complications, while just one experienced a major one. Among the minor neurological complications, the most frequent were quadrantanopia resulting from temporal lobe epilepsy surgeries. As seen in the study by Gooneratne et al., persistent neurological adverse events were observed in 17.2% of their sample. Similarly, quadrantanopia was the most common complication following temporal lobe resections, corresponding to 8.5% of the procedures [8]. Mathon et al. reported that homonymous superior contralateral quadrantanopia occurred in 8.2% of cases. Visual field deficits frequently develop following surgery for mesial temporal lobe epilepsy (MTLE), typically manifesting as contralateral superior quadrantanopia due to injury to the Meyer loop during access to mesial temporal structures [9].

Older age at epilepsy onset was found to be (*p* = <0.001) statistically significant for the presence of surgical complications, regardless of the type of surgery. A meta-analysis by Vary O’Neal et al. highlighted that older age in PWE is associated with up to three times the number of complications [10]. Postoperative hygroma and memory deficits, especially after dominant temporal lobe resection, have been shown to be more common in older patients [11]. Additionally, older patients may have a higher incidence of transient neurological deficits and ongoing surgical complications [12]. Despite these complications, the overall safety profile of epilepsy surgery in older adults is considered acceptable, and advanced age should not hinder the procedure. Comprehensive presurgical evaluation and candidate selection are essential for optimizing outcomes and minimizing these complications.

The association between complications and the type of epilepsy surgery was also evaluated; however, the overall rate of complications did not show statistically significant results, except for visual complications in patients who underwent (*p* = <0.004) temporal lobectomy. Pereira Dalio et al. reported that patients who underwent temporal lobectomy due to temporal lobe epilepsy associated with hippocampal sclerosis (TLE-HS) only presented chronic complications in 5%, with chronic headache being the most common [13]. In comparison with our study, patients who underwent temporal lobectomy only presented cranial nerve deficit, memory disturbances, hemiparesis, and hemianopsia as major neurological complications.

A study conducted by Alanazi G. et al. compared patients who underwent CC and VNS. The complications noted included CSF leaks and swallowing difficulties. The study assessed the procedural outcomes of both interventions and found no statistically significant differences. Notably, no complications were observed with VNS; however, only one patient was included in this group. Further studies with a larger sample size and a more extensive learning curve are necessary to evaluate accurately the complications associated with VNS implantation [14].

The only patient who experienced major neurological complications presented with cranial nerve deficits, memory disturbances, hemiparesis, and hemianopsia. However, no official report regarding probable cause is available. Among the hypotheses proposed by the neurosurgeons in collaboration with the epileptologists, it is suggested that cranial nerve deficits and memory disturbances are likely due to the expected complications of the lobectomy. In contrast, hemiparesis and hemianopsia are suspected to be the result of an intraoperative stroke, which may have caused the concomitance of these two specific complications in this patient.

Most patients underwent temporal lobectomy with amygdalohippocampectomy, reaffirming mesial temporal lobe epilepsy with hippocampal sclerosis as the most frequent cause of DRE in this cohort. Other procedures, such as lesionectomy, CC, or VNS, were chosen based on the location and characteristics of each patient’s epilepsy diagnosis.

According to the classification of epilepsy surgery complications proposed by W.J. Hader et al., neuropsychiatric complications were also considered. However, in the cohort from our institution, a separate study was conducted by González Mille D.D. et al., which provides a more comprehensive evaluation of neuropsychiatric complications [7, 15].

### Risks of seizures versus risks of epilepsy surgery

DRE is associated with high mortality and morbidity in PWE, particularly regarding sudden unexpected death in epilepsy (SUDEP), which accounts for around 3.7 to 9 per 1,000 patient-years [1, 2]. Surgical intervention has been shown to reduce mortality risk substantially. Rheims et al. demonstrated that patients who underwent surgery had nearly a twofold lower risk of death compared to those who did not. Moreover, perioperative mortality for epilepsy surgery was < 1%, and the risk of SUDEP was three times lower in the surgical group [3]. Similarly, Granthon et al. showed that epilepsy surgery reduced all-cause mortality by 30% in PWE, with a mortality rate of 6.0 vs. 8.7 deaths per 1,000 patients annually in the surgical and non-surgical groups, respectively. Among patients who were not operated on or those whose seizures remained uncontrolled, ongoing seizures significantly increased mortality risk (HR 2.1) and the risk of SUDEP (HR 3.5). SUDEP was the leading cause of death, accounting for 30% of all non-surgical cases. Patients who were not operated on had a significantly increased risk of seizures, significantly increased mortality risk (HR 2.1), and the risk of SUDEP (HR 3.5). SUDEP was the leading cause of death, accounting for 30% of all non-cases. Furthermore, 63% of patients achieved seizure freedom following surgery, and long-term benefits persisted over 15 years [4].

When comparing our findings with other studies, it is notable that no deaths were reported among our patients who underwent epilepsy surgery; this may be attributed to the perioperative management protocols, surgical expertise, and a multidisciplinary approach at our institution. Nonetheless, other centers have highlighted the benefits of epilepsy surgery, not only in achieving better seizure control and improving quality of life but also in reducing the risks of SUDEP and overall mortality in PWE. Therefore, it is a safe and viable therapeutic option for patients who meet the appropriate criteria.

### Results in seizure freedom and antiseizure medication post-surgery

In this cohort, Engel 1 outcome was overall seen in 68.5% of patients; this could be explained as almost half of the surgeries consisted of temporal lobectomies, but also represents a reasonable seizure control in surgically treated patients; despite the inclusion of extratemporal procedures and CC which typically achieve lower rates of seizure freedom. As demonstrated by Wiebe et al., after one year, 58% of patients with temporal lobe epilepsy in the surgical group remained free of seizures with impaired awareness [16].

Vít Všianský et al. assessed surgical outcomes based on the procedure type, showing a seizure freedom rate of 64% for resective surgeries, comparable to the results observed in the present study [17]. Yu et al. conducted a large retrospective analysis demonstrating that epilepsy surgery provides sustained seizure control in patients with DRE, with 78% achieving seizure freedom at two years postoperatively. Although this rate declined to 56% by twelve years, the authors suggest this may be due to incomplete resection or secondary epileptogenesis. In Yu et al.’s cohort, all patients remained on ASMs for at least two years, after which medication tapering was individualized. By the five-year follow-up, 65 patients had successfully discontinued ASMs without experiencing seizure recurrence [18]. Likewise, Všianský et al. observed a significant reduction in ASM use following surgery, which may reflect improved seizure control across their cohort. Together, these findings reinforce the effectiveness of epilepsy surgery in reducing seizure burden and decreasing long-term reliance on pharmacological treatment [17, 18].

The meta-analysis conducted by Dong et al. highlights that open surgical procedures, such as anterior temporal lobectomy and lesionectomy, remain highly effective in achieving seizure freedom among patients with DRE. These procedures consistently demonstrated high seizure freedom rates, often comparable to or even surpassing those seen with minimally invasive techniques, especially in cases with well-defined epileptogenic foci, such as temporal lobe epilepsy [19].

#### Barriers to epilepsy surgery in developing countries

It is vital to address the several barriers to epilepsy surgery worldwide, particularly those found in developing countries like Mexico, barriers are multifactorial and include: (a) *Medical factors*: predominantly delayed referral to specialized centers and outdated perceptions of surgical risks; (b) *Patient-related factors*: lack of knowledge about the surgical procedure, preference for less invasive treatments, or unrealistic expectations regarding surgical outcomes; (c) *Healthcare system factors*: common issues include limited access to advanced technologies and specialized equipment, problems with insurance coverage, and prolonged waiting times; (d) *Socioeconomic factors*: racial and economic inequalities, as well as geographical challenges, often hinder access to specialized centers.

Timely access to epilepsy surgery is hindered by a lack of knowledge and outdated practices among general physicians and neurologists regarding the identification of DRE and the benefits of surgery. Misconceptions about surgical risks and cognitive outcomes persist, delaying referrals to specialized centers despite recommendations from the ILAE. Additionally, a preference for new ASMs and non-surgical therapies diverts attention from surgical options. Poor identification of surgical candidates and inadequate communication about the risks and benefits further contribute to the underutilization of epilepsy surgery, exposing patients to uncontrolled seizures and progressive deterioration [20].

Financial and insurance-related constraints remain major barriers to epilepsy surgery, with many patients either lacking adequate coverage or facing protracted administrative processes that delay approval for essential pre-surgical evaluations and procedures. This issue persists even in high-income countries like the United States and Canada, where underinsured patients experience delays in specialist referrals, limited access to surgical assessments, and increased dependence on emergency care. Geographic disparities further exacerbate access, particularly for individuals in rural or underserved areas, where logistical and economic challenges hinder access to specialized epilepsy centers. Additionally, inconsistencies in the availability of advanced diagnostic technologies and specialized expertise contribute to variability in diagnostic protocols and patient selection, leading to unequal care delivery. Overcoming these structural deficiencies will require healthcare policy reforms, broader insurance coverage, and a more equitable distribution of specialized resources to ensure timely and appropriate surgical intervention for patients with drug-resistant epilepsy [20, 21].

In a comparative analysis of the Saskatchewan Epilepsy Program (SEP) in Canada and the NINN in Mexico, the average time from epilepsy diagnosis to surgery was nearly 20 years at both centers, with no statistically significant differences. Specifically, the time from diagnosis to initial referral was 16.9 years at SEP and 18.9 years at NINN. In comparison, the interval from diagnosis to video-EEG telemetry was 18.6 years at SEP and 21.1 years at NINN. Although waiting times from the first consultation with an epileptologist to essential evaluations and surgery were shorter at SEP, they remained prolonged, with patients waiting an additional 25.7 months for surgery at SEP compared to 42.19 months at NINN. These prolonged delays leave patients vulnerable to extended periods of uncontrolled seizures, increased morbidity, and diminished quality of life. The results highlight an urgent need to improve referral processes, ensure adherence to established guidelines, and expand access to specialized epilepsy centers to reduce waiting times and improve outcomes for patients with DRE [22].

The pre-surgical counseling plays a critical role in aligning patient expectations with the realistic outcomes of epilepsy surgery. Ozanne et al., highlighted that while most patients hoped for significant improvements in seizure control and quality of life, many simultaneously experienced profound anxiety regarding potential surgical risks and long-term adverse effects. These concerns included fears of cognitive impairment, speech or vision disturbances, personality changes, and paralysis. Additionally, patients expressed worries about prolonged recovery periods and financial burdens associated with surgery. Despite receiving preoperative counseling, misconceptions about the likelihood and severity of these complications persisted, suggesting that current counseling approaches may not fully address the psychological dimensions of the surgical decision-making process. Therefore, enhancing patient education with a more personalized and comprehensive approach that includes factual information and emotional support is essential. Addressing these concerns effectively may improve patient satisfaction, facilitate informed decision-making, and promote better psychosocial adaptation following surgery [23].

It is worth noting that very few publications worldwide consistently and accurately report complications in epilepsy surgery. Most reports are presented in a very general and heterogeneous manner, as most published articles analyze procedures individually rather than collectively from the perspective of a single center.

### Study limitations

Within the limitations of this study, we identified the need to analyze complications within a more recent time frame. It would be valuable to examine complications over the last decade, as it would allow for comparison concerning the learning curve of new surgical techniques and the use of the latest tools for performing these procedures. Furthermore, it was noted that not all surgical cases of patients with epilepsy are discussed in the epilepsy surgery sessions; for example, some structural epilepsies were not included in the session, which could introduce a bias in the inclusion of patients in this study. This study does not include pathology reports; however, this would be of great interest as it explores an area of opportunity for future research.

According to the classification of epilepsy surgery complications proposed by W.J. Hader et al., neuropsychiatric complications are also considered. However, in the cohort from our specific institution, a separate study was conducted by González Mille D.D. et al., which provides a more comprehensive evaluation of neuropsychiatric complications [7, 15].

It is essential to mention that in this study, our experience does not include procedures such as deep brain stimulation (DBS), hemispherectomy, or phase II approach, such as the use of stereo-electroencephalography (SEEG), which is not available in our Institution then or nowadays. Additionally, although complications related to VNS were included, we only had one patient implanted with the device.

## Conclusion

Despite the low incidence of complications observed in this large LATAM cohort from Mexico, epilepsy surgery remains underutilized due to multiple barriers, limiting access to surgical evaluations for PWE who could benefit from this intervention.

The low accessibility to epilepsy surgery has significant repercussions, as the morbidity and mortality associated with epilepsy far exceed the potential risks and complications of epilepsy surgery when performed by experienced specialists.

Expanding access and decreasing barriers to epilepsy surgical evaluations and interventions are essential to improving patient outcomes and reducing epilepsy-related morbidity and mortality.

## Data Availability

The data contained in this study are the property of Iris E. Martínez Juárez, MD; but can be provided if needed.
